# 5-HT3 antagonist, tropisetron, ameliorates age-related renal injury induced by D-galactose in male mice: Up-regulation of sirtuin 1 

**DOI:** 10.22038/IJBMS.2024.74025.16098

**Published:** 2024

**Authors:** Atefeh Mirshafa, Mohammad Shokati Sayyad, Ebrahim Mohammadi, Fereshteh Talebpour Amiri, Fatemeh Shaki

**Affiliations:** 1 Pharmaceutical Sciences Research Center, Hemoglobinopathy Institute, Mazandaran University of Medical Sciences, Sari, Iran; 2 Ramsar Campus, Mazandaran University of Medical Sciences, Ramsar, Iran; 3 Student Research Committee, Mazandaran University of Medical Sciences, Sari, Iran; 4 Environmental Health Research Center, Research Institute for Health Development, Kurdistan University of Medical Sciences, Sanandaj, Iran; 5 Department of Anatomy, Faculty of Medicine, Molecular and Cell Biology Research Center, Mazandaran University of Medical Sciences, Sari, Iran; 6 Department of Toxicology and Pharmacology, Faculty of Pharmacy, Mazandaran University of Medical Sciences, Sari, Iran

**Keywords:** Aging, Apoptosis, Nephrotoxicity, Oxidative stress, Tropisetron

## Abstract

**Objective(s):**

The kidney ages faster than other organs due to changes in energy metabolism, mitochondrial dysfunction, and oxidative stress. This study looked into the anti-aging effect of tropisetron.

**Materials and Methods:**

D-galactose was administrated subcutaneously in a mouse model for eight weeks in order to induce renal aging. Three separate intraperitoneal doses of tropisetron (1, 3, and 5 mg/kg body weight) were given at the same time. We assessed markers of mitochondrial dysfunction, oxidative stress, and inflammation. Via Real-Time PCR, the expressions of genes linked to aging (SIRT1) and apoptosis (Bax and Bcl-2) were ascertained. In addition, an assessment of histopathological changes, blood urea nitrogen, and creatinine concentrations was done.

**Results:**

In kidney tissue, tropisetron reduces mitochondrial dysfunction and oxidative stress, which are caused by D-galactose-induced overproduction of inflammatory mediators. Additionally, tropisetron demonstrated antiapoptotic activity in renal tissue and augmented the decrease in SIRT1 gene expression associated with D-galactose administration. Besides, tropisetron significantly improved the histological alterations in the renal tissues of aged mice and effectively decreased the elevated levels of creatinine and also blood urea nitrogen.

**Conclusion:**

The results provided additional insight into the effect of tropisetron on renal aging and the underlying mechanisms, particularly through its ability to modulate SIRT1 signaling.

## Introduction

Compared to other organs, the kidney ages more rapidly, and changes related to renal age are known to become elevated in arteriosclerosis, interstitial fibrosis, tubular atrophy, and glomerulosclerosis (1, 2). Growing evidence has demonstrated that oxidative stress and inflammatory responses are possible biological mechanisms in aging. Extreme reactive oxygen species (ROS) generation during senescence can cause cell damage and lead to organ failure. Additionally, ROS overproduction can deplete adenosine triphosphate (ATP) and cause mitochondrial dysfunction, triggering cytochrome c to leak from mitochondria into the cytoplasm and ultimately result in cell apoptosis. As we age, pro-inflammatory cytokines like tumor necrosis factor-α (TNF-α) and interleukin-6 (IL-6) are overexpressed, leading to organ damage and uncontrollably high inflammatory responses (3). In mammals, of the seven known sirtuins, sirtuin 1 (SIRT1) function is the most studied and has been shown to regulate various cellular functions that affect aging and metabolic homeostasis. It also helps protect cells through mitochondrial biogenesis, metabolism, and autophagy regulation in response to redox status and cellular energy. As a result, SIRT1 is assumed to be a potential candidate for regulating renal cell survival against aging (4-6).

As an agent, D-galactose (DG) is mostly utilized for experimental aging induction. DG-induced models of animal aging have similarities with natural aging. DG converts to glucose within the body if used in an insignificant amount, which plays a role in metabolism. Nevertheless, the consumption of huge DG amounts leads to metabolic disorders in cells. This results in oxidative enzyme activity variations, as well as elevated creation of oxidative products and superoxide anions. This, in turn, may lead to oxidative damage in large biological molecule function and structure and thus cause body aging. Aging models are developed through DG administration and are extensively utilized for anti-aging efficacy assessment of anti-oxidant compounds and also for anti-aging health product development (7-9).

Given the complex relationship between aging and renal diseases, anti-oxidative, anti-inflammatory, and anti-apoptotic drugs appear to be a useful tactic for postponing or curing age-related renal diseases. As a 5-Hydroxytryptamine subtype 3 (5-HT3) receptor antagonist, tropisetron is presently demonstrating clinical indications for emesis induced by cancer chemotherapy and post-operative situations. Tropisetron is one of the 5-HT3 receptor inhibitors that recent research has shown to have important anti-inflammatory, immunomodulatory, and anti-oxidative qualities (10, 11). Numerous *in vitro *and* in vivo* studies support these tropisetron properties, For example, tropisetron protects against optic nerve crush injury in rats by suppressing apoptosis (12), mitochondrial dysfunction alleviation via the reduced activity of the nitrergic system in the mouse brain (13), and reduced apoptotic responses and testicular inflammation induced by streptozotocin in rats (14). Tropisetron also reduced experimentally induced cardiac and liver damage in rodent models by acting through antiapoptotic and anti-inflammatory pathways (15). Furthermore, a recent study provided compelling evidence that apoptosis and inflammatory responses play a role in diabetes-induced liver damage and that tropisetron, an anti-oxidant and anti-inflammatory drug, can lessen these effects (16).

We recently demonstrated that tropisetron exhibits neuroprotective impacts within the context of DG-induced senescence in mouse brains and is elucidated by exerting anti-oxidant and anti-inflammatory effects (through various pathways including the SIRT1 signaling activation) (17). In this study, we hypothesized that this drug might ameliorate renal injuries related to aging, which are caused by mitochondrial dysfunction, oxidative stress, inflammation, and programmed cell death via regulation of the SIRT 1/ROS/TNF-α pathway. 

## Materials and Methods

***Animals***

Male Swiss albino mice weighing between twenty and twenty-five grams, at three months of age, were obtained for this study from the Laboratory of Animals Research Center at Mazandaran University of Medical Sciences in Sari, Iran. Ethical protocols established by the Mazandaran University of Medical Sciences Committee of Animal Experimentation were followed throughout the experiment (ethical code: IR.MAZUMS.REC.1396.3084). The mice were housed in an environment maintained at 22±2 °C, with access to proper food and water and a 12:12 hour light cycle. The minimum number of mice required for testing was used, and proper anesthesia procedures were ensured during the experimentally painful procedures (42).

***Drugs and treatments***

In this study, we utilized tropisetron, which we dissolved in a standard saline solution and delivered intraperitoneally (IP), by three different doses of 1, 3, and 5 mg/kg of body weight (BW). DG was also dissolved in a standard saline solution and then injected subcutaneously (s.c). Every solution that was used in this study was created on the day that the experiment took place.

Mice were indiscriminately placed in six equal groupings, with every study group limited to twelve mice. 

Group 1: received normal saline IP, as the vehicle control group (NS) 

Group 2: D-galactose s.c (200 mg/kg body weight) (DG)

Group 3: tropisetron (1 mg/kg BW) IP with DG (200 mg/kg BW) s.c. (T1+DG)

Group 4: tropisetron (3 mg/kg BW) IP with DG (200 mg/kg BW) s.c. (T3+DG)

Group 5: tropisetron (5 mg/kg BW) IP with DG (200 mg/kg BW) s.c. (T5+DG)

Group 6: 20 IU/kg BW of VitE was used as the positive control IP with 200 mg/kg BW of DG s.c (VitE+DG)

Dosages of DG and tropisetron were selected according to previous research and customized during the pre-test period. The study was performed for a period of 56 days (i.e., 8 weeks) (25, 43).

Twenty four hours following the last administration, the mice used in the study were humanely anesthetized using ketamine (80 mg/kg of BW) and xylazine (5 mg/kg of BW). In each group, a total of 6 mice were selected for histopathological assay, determination of blood urea and creatinine concentrations, aging, and apoptosis-related gene expression evaluations. Another six mice per group were utilized for additional tests, including evaluation of oxidative stress, pro-inflammatory, cytokines, and dysfunction markers in the mitochondria.

**Figure 1:**
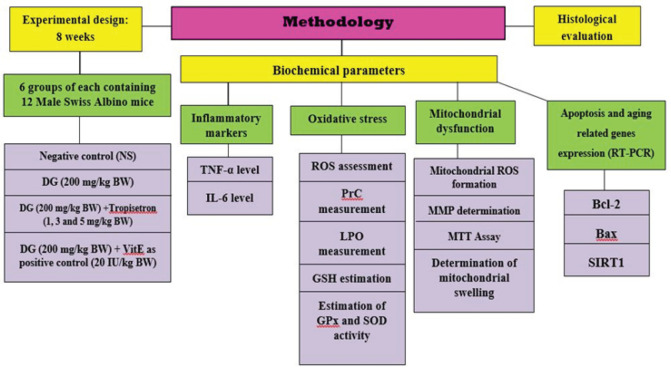
Illustration of the experimental study’s methodology

The kidney sample that was taken for histopathological evaluation was preserved in a solution of formalin (10% w/v), which was kept at room temperature. The sample needed for the RT-PCR (real-time polymerase chain reaction) was stored at -80 °C in an RNA protection solution. The kidney sample for the additional study subjects was then divided, minced, and homogenized using a handheld homogenizer made of glass. The divided tissue portions were utilized for mitochondrial isolation by means of a differential centrifugation technique that we established using succinate dehydrogenase enzyme analysis (44, 45). A flowchart detailing the design of this study is illustrated (Figure 1).

***Total protein assay***

The Coomassie Blue protein binding Bradford method was used to determine protein concentrations (46).

***Markers of oxidative stress***

*Assessment of ROS*

The type of dye we used as a marker to determine the ROS formation in kidney tissue homogenates was 2′,7′- dichlorofluorescein diacetate (DCFH-DA). Using the Shimadzu RF5000U fluorescence spectrophotometer, we determined this conversion at 485 and 520 nm, respectively, for excitation and emission wavelengths. The fluorescent intensity for one milligram of protein was used to determine the ROS level (47).

*Measurement of protein carbonyl (PrC)*

PrC measurement was performed via the spectrophotometric methodology, which is based on guanidine hydrochloride. The protein carbonyl content was determined by measuring absorbance at 365 nm wavelength (48).

*Evaluation of lipid peroxidation (LPO)*

The LPO determination marker used in this study was thiobarbituric acid (TBA), which was a result of malondialdehyde (MDA) production. Using an ELISA reader, the supernatant absorbance at 532 nm was confirmed in order to determine the amount of MDA formation. The standard is Tetramethoxypropane, with the results noted in micromolar (µM) per mg of protein (49).

*Calculating reduced glutathione levels (GSH)*

To evaluate the amount of reduced glutathione within the renal tissue homogenates, we used the following marker, dithio-bis (2-nitrobenzoic acid) (DTNB). The absorbance of the color yellow that was produced was noted at 412 nm on the spectrophotometer (UV-1601 PC). Results pertaining to this were conveyed in µM per milligram of protein (50).

***Measuring the activity of superoxide dismutase (SOD) and glutathione peroxidase (GPx)***

The kidney homogenate was centrifuged for 10 min at 4 °C and 4000 rpm. Following that, the supernatant was collected and utilized, in compliance with manufacturer instructions, to determine the level of GPx and SOD activity in the kidney using standardized kits (ZellBio GmbH assay kits) (50).

***Mitochondrial function assay***

*Measurement of mitochondrial ROS level*

Assessment of ROS levels in the mitochondria was completed using the flow cytometry method with DCFH-DA indicator. To obtain signs, we used a 530-nm band pass filter (FL-1 channel). By using the Flomax software package, we were able to assess the flow cytometry results. Based on 100,000 events, the mean fluorescence intensity was used to make each determination (51).

*Measuring the potential of the mitochondrial membrane (MMP)*

MMP was measured by taking into account and measuring the mitochondria’s intake of the cationic fluorescence probe rhodamine 123. At 535 nm for emission and 490 nm for excitation, a Schimadzou RF-5000U fluorescence spectrophotometer was utilized to track the rhodamine123 fluorescence (52).

*Assessment of mitochondrial function*

By using tetrazolium salt (MTT), we were able to evaluate the level of mitochondrial toxicity. Succinate dehydrogenase, an enzyme in the mitochondria, makes this yellow marker into a purple formazan. Dimethyl sulfoxide is then used to dissolve these formazan crystals, with its rate of absorbance being measured by an ELISA reader noted as 570 nm in wavelength (53).

*Measures the swelling of the mitochondria*

Two techniques were used to measure the variations in the swelling of mitochondria that were separated from the kidney tissue: light scattering and flow cytometry. By using an ELISA reader (Tecan, Rainbow Thermo, Austria) to track light scattering at 540 nm at a temperature of 30 °C, the swelling of mitochondria was determined using the light scattering method. Low-angle light scattering was used to analyze mitochondrial swelling in the flow cytometry method. Analysis was conducted using a flow cytometer (Partec, Deutschland) fitted with a 488-nm argon ion laser. Light scattering and mitochondrial fluorescence were measured for a minimum of 100,000 counts utilizing the Flomax program (Partec, Deutschland). In the FL-1 channel, fluorescence signals were acquired using a 530-nm band pass filter (54).

***Inflammation evaluation: TNF-α and IL-6 levels***

The discovery of IL-6 and TNF-α quantities in the kidney samples was conducted with enzyme-linked immunosorbent assay specific to mice ELISA kits. The absorption in the final dyed output was then quantified as the primary wavelength at 450 nm, with a wavelength reference of 620 nm. The following inflammatory mediator’s levels were stated as pg/mg (55).

***Real-time PCR: B-cell lymphoma 2 (Bcl-2), Bcl-2-associated X (Bax), and SIRT1 gene expression***

Kidney tissue sample that had been stored in an RNA protector solution at -80 °C was utilized for the complete extraction of the RNA. Kidney tissue samples were processed via a microtube using the Hybrid-R™ total RNA isolation kit, following the manufacturer›s instructions. The integrity of the extracted RNA was checked using particular quantitative and qualitative techniques. The quantity measurement check was made by mixing 97 μl of diluted water with 3 μl RNA; the absorption rate was determined at 260 and 280 nm using a spectrophotometer. An agarose gel was used for electrophoresis during the quality measurement checks. Our ReverAid First Strand cDNA Synthesis Kit from Thermo Scientific was used to create cDNA. Using particular primers for Bax, Bcl-2, and SIRT1 genes, we performed RT-PCR on all study samples (Table 1). Actin was used as the gene of reference by the Corbett machine. The RT-PCR program of Bcl-2, SIRT1, and Bax, as well as actin genes are noted in Table 2 (56, 57).

***Renal histopathology and measurement of creatinine and blood urea nitrogen (BUN)***

For the examination of histopathology, in consideration of the possible influence of tropisetron and DG on the samples of collected renal tissue, we set the samples for 24 hr in a formalin solution of 10% (w/v). After processing samples, they were then embedded in paraffin by following a standardized method. For the evaluation of possible renal damage, the samples with a 5 µm width were then stained using both Hematoxylin and Eosin (H & E). Using x400 magnification, the collected segments were evaluated for the degree of kidney matter by a histologist. To ensure study reliability, the histologist was blinded as to which samples were collected from the treatment group. The histological evaluation was conducted using an Olympus light microscope (58). 

Following experimental treatment, blood samples were taken from the hearts of the mice. By centrifuging at 3000 rpm for 10 min at 4 °C, the plasma was separated.  An automatic biochemistry analyzer (RT-2100, Rayto Shenzhen Rayto Life Science Co., China), as per the guidelines provided by the assay kit providers, was used to determine the BUN and Cr concentrations in serum (43).

***Statistical analyses***

The results were demonstrated as Mean±SEM. Version 16 of SPSS software was used to complete all statistical analyses for this study. We conducted a one-way ANOVA and a Turkey Test, with significance set at *P*<0.05. 

By means of the Ct variations and 2^-ΔΔcT^ (Ct test- Ct reference) formula, and in using actin as a reference gene, we analyzed the gene expressions of SIRT1, Bcl-2, and Bax genes. We finished our assessment of the validity of the RT-PCR by creating a dilution from the research samples and creating a standard curve. The t-test, ANOVA, and Kruskal-Wallis tests were used in the data analysis process using SPSS software version 16. We computed the mean, maximum, average, and minimum for every research data set. For us, statistical significance was defined as *P*<0.05.

## Results

***Indicators of oxidative stress***

*Reactive oxygen species level*

In comparison with the normal saline group, we observed that the study group that was administered DG had significantly (*P*<0.001) raised production of ROS. We also noted that when there was a co-treatment with tropisetron doses, there was a significant decrease in the DG-induced formation of ROS when measured against the DG group (*P*<0.001). Furthermore, we found that co-administration of VitE was able to considerably (*P*<0.001) lower the DG-induced ROS production (Table 3).

*Protein carbonyl concentration*

Table 3 shows that, when compared to the study control group, the PrC level increased after DG chronic administration (*P*<0.001). Also, in comparison to the DG group, we discovered that co-treating aged mice renal samples with tropisetron at all doses and VitE could significantly (*P*<0.001) lower PrC concentrations.

*Lipid peroxidation*

We found that when compared to the control group, a chronic injection of DG was able to significantly (*P*<0.001) elevate the MDA levels, which is the main by-product of the peroxidation of lipid molecules. We also observed that when co-treated with tropisetron there was a significant (*P*<0.001) reduction in DG-induced LPO at all doses. Moreover, we identified an identical outcome when co-administering VitE (Table 3). 

*Glutathione content*

Table 3 clearly shows that, when DG was administered, there was a significant (*P*<0.001) decrease in the glutathione content when compared to the control group. We discovered that there was a noteworthy (*P*<0.001) reversal in the DG-induced GSH oxidation at all three doses of co-treatment with tropisetron. Furthermore, the VitE co-treatment and tropisetron study groups showed similar results.

*SOD and GPx activity*

We discovered that treating with DG alone led to significantly (*P*<0.001) reduced GPx and SOD activity in the renal tissue when compared to the control group. In contrast, when tropisetron is administered chronically in addition to VitE, we noted a significant (*P*<0.001) reduction of oxidative damage that was signified through GPx and SOD level restoration, when compared with the DG group (Table 3). 

***Mitochondrial function assay***

*Mitochondrial ROS level*

**Figure 2:**
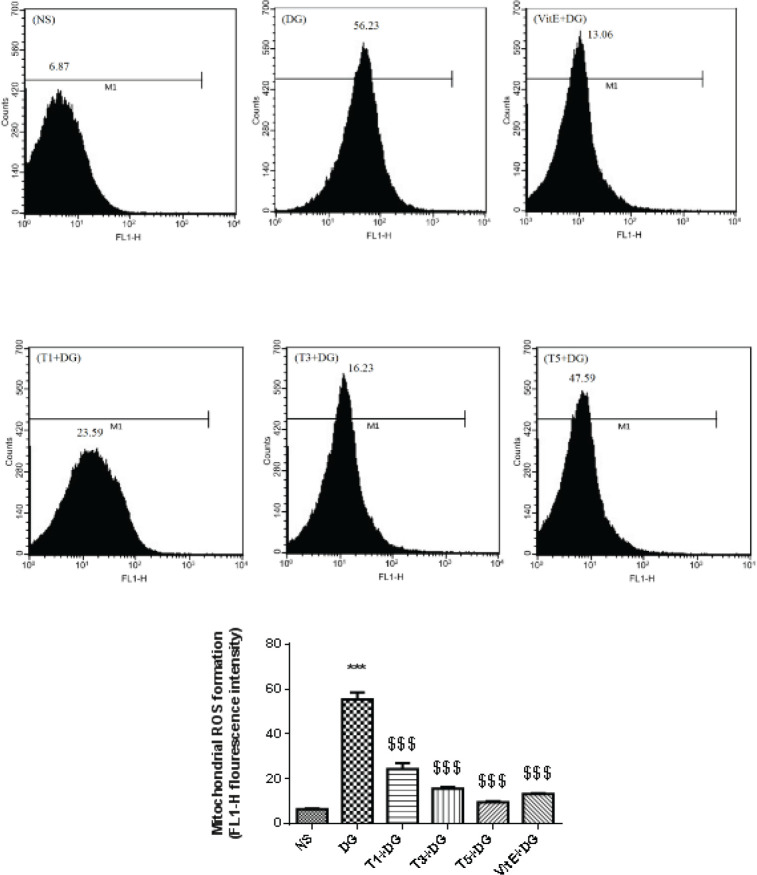
Effects of treatment on the generation of reactive oxygen species (ROS) in mitochondria isolated from the kidney tissue of mice

In comparison to the control group, we noted that administering DG led to a significant (*P*<0.001) increase in the formation of ROS, this is illustrated in Figure 2. Compared with the DG study group, the group that received tropisetron treatment had a notable (*P*<0.001) decrease in ROS production caused by DG. Moreover, we found that giving VitE significantly (*P*<0.001) reduced the amount of ROS produced by DG.

*Mitochondrial membrane potential*

**Figure 3:**
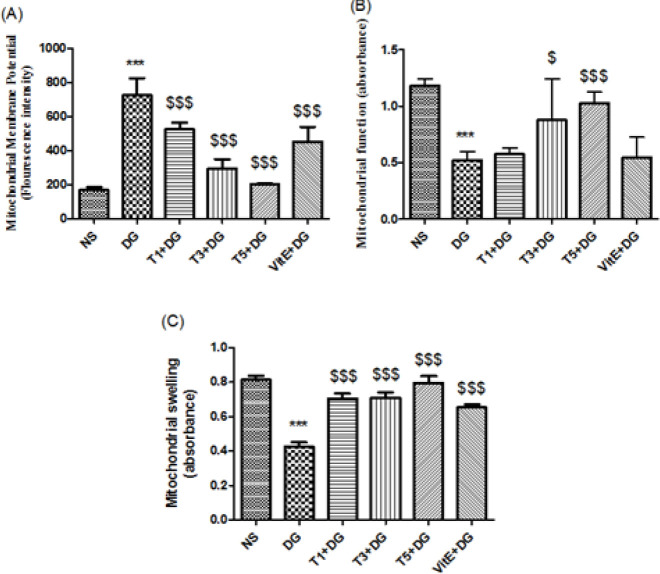
Treatments’ effects on mice kidney-isolated mitochondria’s (A) mitochondrial membrane potential, (B) mitochondrial function, and (C) mitochondrial swelling. The data displayed are the Mean ± SD of six mice per group

A sure sign of apoptosis and the dysfunction of the mitochondria is the opening of mitochondrial permeability transition (MPT), which indicates MMP collapse. In this study, we noted that DG induced significant (*P*<0.001) MMP collapse; however, this was reversible through co-treatment of tropisetron at all three doses. In addition, in Figure 3A it is evident that VitE was able to noticeably prevent DG-induced mitochondria MMP collapse (*P*<0.001).

*Mitochondrial function*

Compared with the control group, we established that by administering DG there was a significant (*P*<0.001) decrease in function of the mitochondria as evident in the mitochondrial renal tissue (Figure 3B). Also, in comparison with DG-treated mice, we observed that tropisetron was able to significantly (*P*<0.001) decrease mitochondrial toxicity that was induced by DG, at doses of both 3 and 5 mg per kg of BW. On the other hand, the mitochondrial function of aged mice given 1 mg per kg of BW tropisetron and VitE improved more, albeit not significantly.

*Mitochondrial swelling*

**Figure 4:**
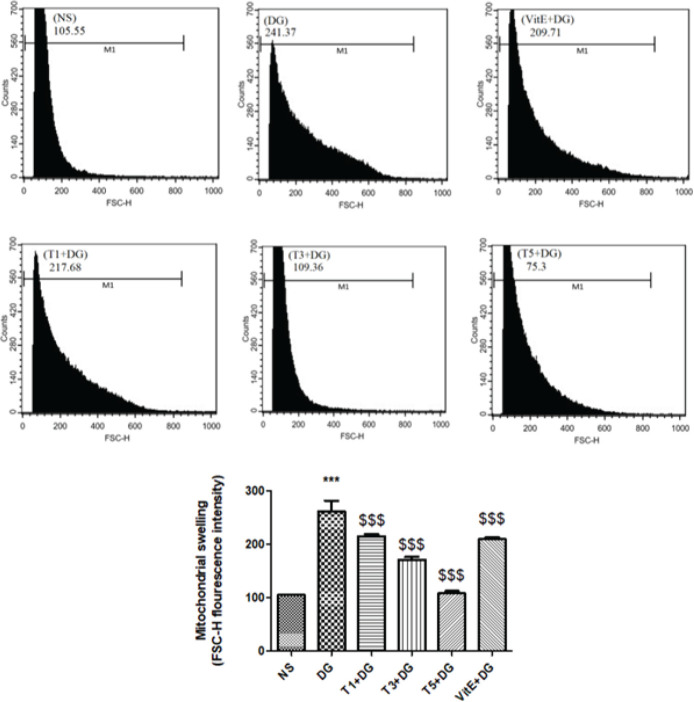
Treatment effects on swelling of mitochondria in kidney-isolated mitochondria of mice

Compared to the control group, a significant increase (*P*<0.001) in mitochondrial swelling resulted from administering DG. Additionally, the 3 different doses of tropisetron along with VitE significantly lessened the 540 nm of absorbance in the kidney mitochondria that were treated with DG (Figure 3C). Figure 4 illustrates that in this study the first method findings were verified by the outcome noted by the flow cytometry method.

***Inflammation: TNF-α and IL-6 levels***

TNF-α and IL-6 levels in the DG-treated group were remarkably higher (*P*<0.001) than in the control group when compared to the NS grouping. Tropisetron and VitE co-administration, however, clearly prevented the IL-6 increases (*P*<0.001). Furthermore, compared to the aged mice in the DG group, VitE and two doses of tropisetron (3 and 5 mg/kg BW) were demonstrated to significantly lower the pro-inflammatory mediator, TNF-α (Table 4).

***Apoptosis: genes expressions of Bcl-2 and bax***

**Figure 5:**
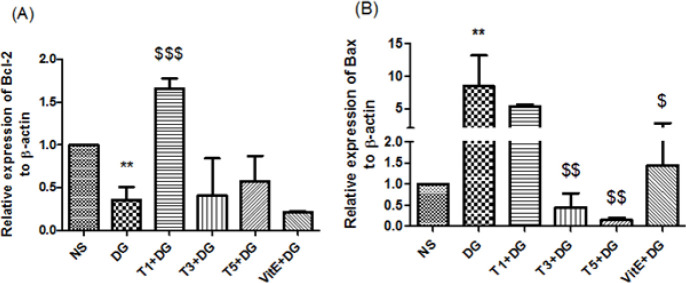
Treatment-induced changes in the expression of (A): Bcl-2 and (B): Bax genes in mouse kidney tissue

Although not statistically significant, the RT-PCR analyses indicate that the Bcl-2 expression levels for the DG group declined in comparison to the study controls. As a result of concurrently treating with tropisetron, there has been a noticeable rise in the expression levels of Bcl-2, but solely at 1 mg per kg of BW dose (Figure 5A). additionally, we observed that the kidney sample from the DG group had higher levels of Bax gene expression than that of the NS group. On the other hand, compared to the DG group, Bax expression was down-regulated by tropisetron at the experimental doses of 3 and 5 mg/kg BW as well as VitE (Figure 5B).

***SIRT1 gene expression***

**Figure 6:**
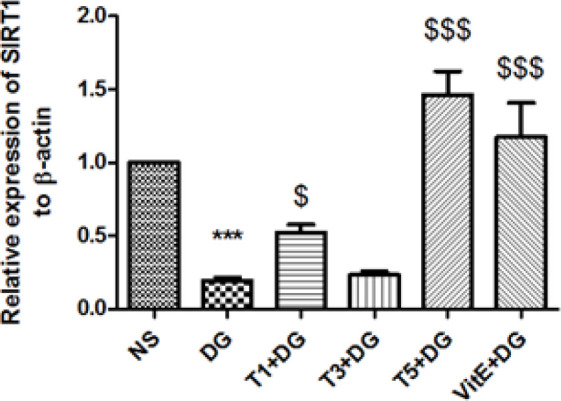
Alterations in SIRT1 gene expression in kidney tissue from mice caused by various treatments

The SIRT1 gene, which is a longevity factor, showed significantly lower expression levels in the DG group when compared to the control group, according to RT-PCR analysis. Co-administration of VitE and tropisetron at two doses (1 and 5 mg/kg BW) increased SIRT1 gene expression (Figure 6).

***Renal histopathological changes and concentrations of BUN and Cr***

**Figure 7:**
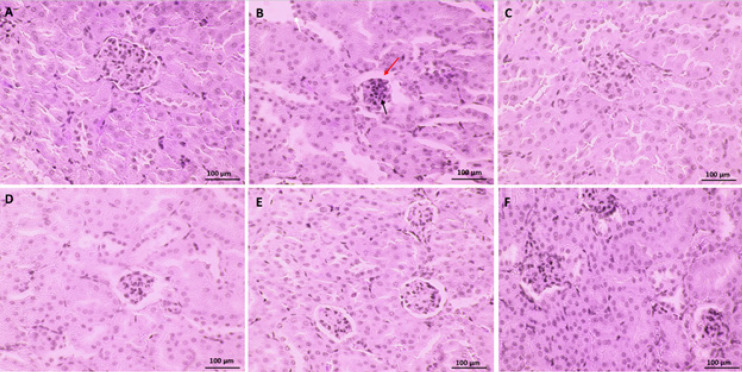
Effects of Tropisetron and D-Galactose on the mice kidney’s histological architecture were depicted in photomicrographs as follows: (A) Normal saline; (B) D-Galactose (200mg/kg) (C) VitE+ D-Galactose, (D) Tropisetron 1mg/kg and D-Galactose, (E) Tropisetron 3mg/kg and D-Galactose, and (F) Tropisetron 5mg/kg and D-Galactose

Figure 7 displays the kidney photomicrographs for each group. In the control group, the tubular epithelial cells and glomeruli in the mice’s kidney displayed normal morphological appearance. DG alone group showed necrotic nephrocyte nuclei and Kupffer cell proliferation. Co-treatment with VitE and tropisetron induced relatively good recovery in renal tissue compared to the DG alone group. 

BUN and Cr levels were significantly (*P*<0.001) elevated in the DG group as an indicator of renal dysfunction in contrast to the control group. Table 5 shows that after co-treatment with all doses of tropisetron and VitE, the elevated levels of BUN and Cr were significantly suppressed (*P*<0.001).

## Discussion

Alterations through senescence are a critical risk factor in inducing different diseases in body organs, particularly the kidney which ages more quickly compared to the other organs (18). On the other hand, there are lots of materials and drugs such as alcohol, methamphetamine, and lipopolysaccharides that can damage the kidney and induce renal injury which is similar to aging (19). It is difficult to develop anti-aging interventions because the mechanisms underlying senescence are still unknown. However, some theories suggest that oxidative stress caused by free radicals, changes in mitochondrial permeability, apoptosis, inflammatory pathways, DNA damage, protein aggregation, and misfolding are the mechanisms underlying aging (20). We can accelerate senescence in rodents with chronic administration of DG. When DG reaches high levels, galactose oxidase converts it to aldose and hydroperoxide. This reaction ends in oxygen-derived free radicals (21). ROS can attack macromolecules of cells including DNA, lipids, and proteins which leads to oxidative damage, mitochondrial dysfunction, apoptosis, and inflammatory reactions (22). These consequences are similar to the aging process. Therefore, chronic DG injection in mice is a well-established model for anti-aging research. In previous research, it was revealed that chronic administration of DG induces oxidative stress and inflammatory reactions in mice that ends in renal injury and dysfunction (19). Our investigation supported earlier findings and demonstrated that long-term DG administration can cause apoptosis, mitochondrial oxidative stress, inflammation, and down-regulation of SIRT1 gene expression in renal tissue. Anti-oxidative pharmaceutical compounds can reverse or delay age-associated impairments by regulating molecular and cellular pathways related to oxidative damage, inflammation, and apoptosis (23). Furthermore, they may induce mitochondrial stabilization and chelating activities (24). The protective qualities of 5-HT3 receptor antagonists *in vivo *and* in vitro* have been studied recently. Tropisetron is one of these 5-HT3 blockers that was first prescribed as an antiemetic medication, particularly during chemotherapy and following surgery. According to tropisetron’s proposed anti-inflammatory, anti-apoptotic, and anti-oxidant properties (25-28), we selected this 5-HT3 antagonist as our preventive medication and as a possible therapeutic approach to mitigate kidney damage during DG-induced kidney aging in mice. As an anti-aging protein, SIRT1 is the most intensively studied sirtuin among the seven sirtuins in mammals. By using the coenzyme nicotinamide adenine dinucleotide (NAD^+^), target proteins can be deacetylated with SIRT1. Therefore, cellular energy metabolism and redox state are linked with SIRT1 through different signaling pathways (29). There is a close relation between SIRT1 expression and ROS concentration. According to recent research, increased ROS level is capable of controlling SIRT1 enzyme activity both directly and indirectly. For instance, ROS is able to perform SIRT1 activity inhibition via oxidative modification on its cysteine residues. Besides, decreased SIRT1 activity triggers inflammatory responses by raising nuclear factor kappa B (NF-κB) signaling. Two hallmarks of aging are a decrease in autophagy and a low-grade inflammatory phenotype, which are caused by the SIRT1 and ROS signaling crosstalk (30). Therefore, the elevation in SIRT1 activity in the kidney may be a novel therapeutic strategy to protect kidneys from histological and functional alterations through aging-induced diseases. 

Some studies prove DG can decline the expression level of SIRT1 protein (31, 32). The present study examined the expression of the SIRT1 gene in the renal tissue of aged mice induced by DG following co-treatment with tropisetron. The findings demonstrated that in the DG group, tropisetron and VitE increased SIRT1 gene expression. Additionally, tropisetron increased SIRT1 expression more than VitE, indicating that tropisetron provides robust protection against DG-induced alterations such as inflammation, mitochondrial oxidative stress, and programmed cell death. Nevertheless, the precise pathway responsible for SIRT1’s renoprotective effect remains unclear. The cellular homeostasis system is prone to a greater number of errors, such as erroneous protein quality control and energy metabolic regulation, with the aging of an organism. Among them, mitochondrial defects are considered the critical components of the aging process as well as several diseases related to age. ROS overproduction and oxidative stress that increase with aging are promoted by mitochondrial impairments. An increased level of aberrant protein aggregates and dysfunctional organelles accumulates when ROS production rises. This, in turn, activates cellular receptor systems and inflammatory responses, which deal with molecular patterns related to cell injury. Moreover, various survival mechanisms exist that can deal with dangers related to stress by enhancing housekeeping efficiency. SIRT1 is capable of regulating some survival functions by deacetylating numerous critical transcription variables, including variables that are able to control ROS production. As it appears, increased ROS levels as well as oxidative stress can control SIRT1 activity (32, 33).

Liu *et al.* presently provided evidence that through oxidative damage suppression, tropisetron protects against acetaminophen-induced hepatotoxicity by reducing MDA, raising GSH levels, and stimulating SOD activity (25).

Rahimian *et al.* showed that tropisetron had neuroprotective effects and decreased the apoptotic responses induced by amyloid-beta *in vivo* (34). In addition, the anti-apoptotic effect of tropisetron in the heart and liver has gained attention in other animal studies (15, 16). Apoptosis significantly affects the aging process. The renal cell apoptosis is reported to be closely connected to pro-apoptotic genes such as Bim and Bax (Bcl-2-like protein 11) as well as anti-apoptotic genes like Bcl-Xl and Bcl-2 (Bcl-2-like protein 1) (35). Research demonstrated that tropisetron co-treatment inhibited DG-induced apoptosis by increasing Bcl-2 gene expression and decreasing Bax gene expression. It makes sense that reducing ROS formation and SIRT1 up-regulation by tropisetron would result in lower expression of Bax and higher expression of Bcl-2 given the ROS overproduction and SIRT1 down-regulation caused by DG. In summary, tropisetron inhibits the mitochondrial apoptotic pathway, which may result in renoprotection. According to our findings, repeated DG treatment may significantly hasten the senescent process and cause renal dysfunction, as evidenced by histopathological damage and elevated BUN and Cr levels. Nevertheless, tropisetron co-treatment may be able to successfully reverse these deteriorating changes in renal tissue, consistent with findings related to apoptosis. This may point in part to tropisetron’s renoprotective action against renal impairments and the damage that DG causes to kidney tissue during accelerated senescence. Numerous studies have demonstrated that aging activates the signaling of NF-κB, which is responsible for inducing inflammatory responses (36). Both SIRT1 and oxidative stress have the known ability to control the signaling of the NF-κB pathway (32). Oxidative damage has the potential to trigger renal NF-κB receptors and trigger the release of proinflammatory cytokines like TNF-α and IL-6, in addition to causing direct cell damage. Normal kidney function may be hampered by an overabundance of these mediators. However, because SIRT1 is a strong inhibitor of NF-κB signaling, it can reduce inflammation (32). So we set out to investigate if the anti-oxidant properties of tropisetron and the up-regulation of the SIRT1 gene could lessen the release of inflammatory mediators in the mouse kidneys caused by DG. In a variety of experimental models, prior research has demonstrated that tropisetron can reduce the amounts of NF-κB and inflammatory cytokines like TNF-α and il-6 (34, 37, 38). Tropisetron alleviated experimentally induced liver and heart damage in rodent models through anti-inflammatory mechanisms (15). Moreover, Amini and colleagues, demonstrated how tropisetron’s down-regulation of TNF-α and IL-6 levels had an anti-inflammatory effect on diabetes-induced liver abnormalities (16). Renoprotective effects of tropisetron were exhibited in different experimental models via assessment of different mechanisms. For example, Zirak *et al.* administrated twice daily for three days (3 mg/kg, IP) 5-HT3 antagonists (granisetron, ondansetron, and tropisetron) for defense against nephrotoxicity in mice caused by cisplatin. Interestingly, tropisetron improved renal dysfunction by reducing the expression of oxidative stress and inflammatory markers. Ondansetron and granisetron, two more 5-HT3 antagonists, did not, however, demonstrate any positive effects (39). Researchers demonstrated how tropisetron, at a dose of 3 mg/kg for two weeks, attenuated renal fibrosis by regulating TGF-β1, p53, and the expression of extracellular matrix metalloproteinases. This treatment effectively mitigated the renal fibrosis associated with diabetic nephropathy (40). According to an additional study, tropisetron may reduce oxidative damage and modify the levels of SIRT1, FOXO3a, and claudin-1, which could lessen renal damage brought on by diabetic nephropathy (41). In our investigation, co-administration of tropisetron at three different experimental doses markedly inhibited the overproduction of IL-6 and decreased the rise in TNF-α in the kidneys of mice at doses of three and five mg/kg body weight.

Mice were randomly divided into six experimental groups with 12 mice each, as follows: control group, D-galactose group, D-galactose mice treated with tropisetron (1,3 and 5 mg/kg) and positive control (D-galactose mice treated with VitE). After for 8 weeks treatment, the kidney tissues were separated and divided into 3 parts. One part was fixed in 10% buffered formalin for histopathological investigation. The other 2 parts were stored at −80 °C for biochemical and molecular analyses.

**Table 1:** Primer sequences of Bcl-2, Bax, and SIRT1 genes, along with β-actin as a housekeeping gene in mice

Gene	Forward primers	Reverse primers
Sirt1	ACGCTGTGGCAGATTGTTAT	GCAAGGCGAGCATAGATA
Bcl-2	TCCCGCCTCTTCACCTTTCAG	GTGTTTCCCCGTTGGCATGAG
Bax	CGGCGAATTGGAGATGAACTG	GCAAAGTAGAAGAGGGCAACC
β-actin	GTGACGTTGACATCCGTAAAGA	GCCGGACTCATCGTACTCC

**Table 2:** Real-Time PCR program in kidney tissue of mice

Step	Temperature	Time	Number of cycles
**UNG (uracil-N-glycosylase) pre-treatment**	50°C	2 min	1
**Initial denaturation**	95°C	10 min	1
**Denaturation**	94°C	15 sec	45
**Annealing**	60°C	30 sec	
**Extension**	72°C	30 sec	

**Table 3:** Treatment effects on oxidative stress biomarkers in kidney tissue of mice

Groups	ROS (fluorescence intensity)	PrC (mM)	LPO (μM)	GSH (μM)	GPx activity (U/mg protein)	SOD activity (U/mg protein)
NS	15.94±4.01	0.07±0.01	15.71±3.77	313.52±7.38	354.47±56.56	22.66±1.99
DG	118.26±20.16^***^	0.61±0.03^***^	90.78±4.92^***^	106.09±14.4^***^	46.11±32.19^***^	7.06±2.74^***^
T1+DG	45.53±8.72^$$$^	0.21±0.03^$$$^	19.002±2.99^$$$^	295.83±8.14^$$$^	213.26±77.07^$$^	15.8±1.22^$$$^
T3+DG	32.18±4.14^$$$^	0.19±0.02^$$$^	17.17±3.66^$$$^	309.87±10.53^$$$^	230.55±76.29^$$$^	15.94±0.68^$$$^
T5+DG	24.54±3.56^$$$^	0.14±0.01^$$$^	17.05±3.38^$$$^	311.99±5.72^$$$^	299.71±81.59^$$$^	16.32±1.12^$$$^
VitE+DG	50.11±8.99^$$$^	0.19±0.04^$$$^	22.89±4.22^$$$^	291.38±5.76^$$$^	210.37±63.22^$$^	15.79±2.67^$$$^

Data are Mean ± SD of six mice in each group

The NS group was given normal saline; the DG group received 200 mg/kg of DG alone; the T+DG groups received 200 mg/kg BW of DG and 1, 3, and 5 mg/kg BW of tropisetron concurrently; and the VitE+DG group received 200 mg/kg BW of DG and 20 IU/kg BW of VitE concurrently

NS: Normal Saline, DG: D-galactose, T: Tropisetron

***: *P*<0.001, significantly different from the NS group. $$<0.01, $$$: *P*<0.001, significantly different from DG group

DCFH-DA was used in flow cytometry experiments, as detailed in the “Materials and Methods” section. The intensity of DCF’s fluorescence is FL1. The three animals in each group are represented by the mean ± standard deviation. The NS group recieved Normal Saline, the DG group received only 200 mg/kg of D-galactose; the T+DG groups received 200 mg/kg D-galactose and tropisetron at doses of 1, 3, and 5 mg/kg simultaneously; the VitE+DG group received 200 mg/kg D-galactose and VitE at a dose of 20 IU/kg simultaneously. ***: *P*<0.001, significantly different from the Normal Saline group. $$$: *P*<0.001, significantly different from the D-Galactose group.

The NS group was given normal saline. The DG group received 200 mg/kg of D-Galactose alone; the T+DG groups received 200 mg/kg of D-Galactose and 1, 3, and 5 mg/kg of tropisetron concurrently; the VitE+DG group received 200 mg/kg of D-G and 20 IU/kg of VitE concurrently.

***: *P*<0.001, significantly different from the normal saline group. $<0.05, $$$: *P*<0.001, significantly different from the D-Galactose group

Flow cytometry was used to conduct the experiments, as stated in the “Materials and Methods” section. The three animals in each group are represented by the mean ± standard deviation of the data.

While the NS group was given normal saline. The DG group received only Galactose (200 mg/kg BW). The T+DG groups received D-Galactose (200 mg/kg BW) and tropisetron at doses of 1, 3, and 5 mg/kg simultaneously, the VitE+DG group received both D- Galactose (200 mg/ kg) and VitE at a dose of 20 IU/kg.

***: *P*<0.001, significantly different from the NS group. $$$: *P*<0.001, significantly different from the DG group

**Table 4:** Treatment effects on inflammatory response biomarkers in kidney tissue of mice

Groups	TNF-α (pg/ml)	IL-6 (pg/ml)
NS	82.62±19.29	13.68±1.25
DG	276.89±47.004^***^	54.24±2.76^***^
T1+DG	240.54±22.16	23.22±0.91^$$$^
T3+DG	215.12±18.48^$^	20.07±1.63^$$$^
T5+DG	114.81±30.2^$$$^	16.65±2.91^$$$^
VitE+DG	257.31±29.58	22.3±3.29^$$$^

TThe NS group was given normal saline. The DG group received only D-Galactose 200 mg/kg; the T+DG groups received 200 mg/kg of D-Galactose and tropisetron at doses of 1, 3, and 5 mg/kg simultaneously; the VitE+DG group received 200 mg/kg of D-Galactose and VitE at a dose of 20 IU/kg simultaneously.

***: *P*<0.001, significantly different from the Normal saline group. $<0.05, $$$: *P*<0.001, significantly different from the D-Galactose group

An internal standard within the study was β-actin. Experiments were repeated three times. The information is based on the mean ± standard deviation of three animals per group.

The NS group was given normal saline; the DG group was given 200 mg/kg of D-Galactose alone; the T+DG groups were given 200 mg/kg of D-Galactose and 1, 3, and 5 mg/kg of tropisetron at the same time; and the VitE+DG group was given 200 mg/kg of D-Galactose and 20 IU/kg of VitE at the same time.

**: *P*<0.01, significantly different from the Normal saline group. $<0.05, $$: *P*<0.01, $$$: *P*<0.001, significantly different from D-Galactose group.

An internal standard within the study was β-actin. The information is based on the mean ± standard deviation of three animals per group.

While the NS group was given normal saline. The DG group received only D-Galactose (200 mg/kg). The T+DG groups received D-Galactose (200 mg/kg) and tropisetron at doses of 1, 3, and 5 mg/kg simultaneously, the VitE+DG group received both D-Galactose (200 mg/ kg BW) and VitE at a dose of 20 IU/kg.

***: *P*<0.001, significantly different from the Normal saline group. $<0.05, $$$: *P*<0.001, significantly different from the D-Galactose group.

**Table 5:** Treatment effects on the levels of creatinine and urea in the blood serum of mice

Groups	Urea (mg/dl)	Creatinine (mg/dl)
NS	33.83±3.19	0.37±0.05
DG	57.67±2.16^***^	1.65±0.36^***^
T1+DG	43.83±3.43^$$$^	0.4±0.06^$$$^
T3+DG	42.33±5.32^$$$^	0.4±0.11^$$$^
T5+DG	40.67±3.14^$$$^	0.38±0.13^$$$^
VitE+DG	44.83±3.43^$$$^	0.4±0.09^$$$^

The NS group received Normal saline; The DG group received only D-Galactose at the dose of 200 mg/kg of D-Galactose; T+DG groups received D-Galactose (200 mg/kg BW) and tropisetron at doses of 1, 3, and 5 mg/kg, simultaneously; The VitE+DG group received D-Galactose (200 mg/kg BW) and VitE at the dose of 20 IU/kg, simultaneously.

***: *P*<0.001, significantly different from the Normal saline group. $$$: *P*<0.001, significantly different from the D-Galactose group.

Notice the epithelial desquamation (white arrow), glomeruli atrophy (black arrow), and dilation of Bowman’s space (red arrow) in the DG group. H&E staining (Mag: ×40), Scale bar =100

## Conclusion

Taken together, the findings from this study validate the hypothesis that tropisetron can have an anti-aging effect on the kidney. In addition, the suggested protective mechanisms of tropisetron include ameliorating mitochondrial oxidative stress, protecting renal tissue from apoptotic damage, suppressing inflammation, and up-regulating the SIRT1 gene expression. The up-regulation of SIRT1 can modulate the various pathways involved in the pathogenesis of aging. Further research is recommended to explore other *in vivo *and* in vitro* aging models and molecular targets. This could lead to the development of a novel approach to protect against renal impairments induced by senescence.

## Authors’ Contributions

Conceptualization, F Sh, Methodology, A M, F Sh, E M, F TA, Formal analysis and investigation: F Sh, E M, F TA, A M Writing - original draft preparation: M Sh and A M Writing - review and editing: F Sh, M Sh, A M Funding acquisition and Supervision: F Sh. The published version of the manuscript has been read and approved by all authors.

## Funding Statement

This study was supported by Mazandaran University of Medical Sciences, Sari, Iran (Grant number: 3084) (ethical code IR.MAZUMS.REC.1396.3084).

## Institutional Review Board Statement

The animal experiments were approved by the Institutional Committee for Animal Care and Use at the Mazandaran University of Medical Sciences, Sari, Iran, and carried out in accordance with the Laboratory Animal Guideline of Welfare and Ethics with ethical code (IR.MAZUMS.REC.1396.3084).

## Conflicts of Interest

The authors of this paper declare that they have no conflicts of interest associated with this paper. 
